# 1Identification of genes differentially expressed in the embryonic pig cerebral cortex before and after appearance of gyration

**DOI:** 10.1186/1756-0500-3-127

**Published:** 2010-05-05

**Authors:** Karsten B Nielsen, Mogens Kruhøffer, Ida E Holm, Arne L Jørgensen, Anders L Nielsen

**Affiliations:** 1 Department of Human Genetics, University of Aarhus, The Bartholin Building, DK-8000 Aarhus C, Denmark; 2 Department of Molecular Medicine (MOMA), Molecular Diagnostic Laboratory, Aarhus University Hospital, Skejby Sygehus, DK-8200 Aarhus N, Denmark; 3 Department of Pathology, Aalborg Hospital, Aarhus University Hospital, DK-9000 Aalborg, Denmark

## Abstract

**Background:**

Mammalian evolution is characterized by a progressive expansion of the surface area of the cerebral cortex, an increase that is accompanied by gyration of the cortical surface. The mechanisms controlling this gyration process are not well characterized but mutational analyses indicate that genes involved in neuronal migration play an important function. Due to the lack of gyration of the rodent brain it is important to establish alternative models to examine brain development during the gyration process. The pig brain is gyrated and accordingly is a candidate alternative model.

**Findings:**

In this study we have identified genes differentially expressed in the pig cerebral cortex before and after appearance of gyration. Pig cortical tissue from two time points in development representing a non-folded, lissencephalic, brain (embryonic day 60) and primary-folded, gyrencephalic, brain (embryonic day 80) were examined by whole genome expression microarray studies. 91 differentially expressed transcripts (fold change >3) were identified. 84 transcripts were annotated and encoding proteins involved in for example neuronal migration, calcium binding, and cytoskeletal structuring. Quantitative real-time PCR was used to confirm the regulation of a subset of the identified genes.

**Conclusion:**

This study provides identification of genes which are differentially expressed in the pig cerebral cortex before and after appearance of brain gyration. The identified genes include novel candidate genes which could have functional importance for brain development.

## Findings

The complex architecture of the mammalian cerebral cortex is a consequence of the highly organized movement of neuronal cells. All neurons populating the six layered cerebral cortex undergo mitosis in distant compartments and achieve their final position following migration [1]. This migration is coordinated to obtain specific laminar position, orientation, and connections with other neurons. The number of neurons is dramatically increased throughout mammalian evolution but the expansion in the radial direction is relative limited, and the thickness of the cortex is relative constant [1,2]. The main expansion is observed in the tangential direction which is the most variable and distinctive part of the central nervous system [3]. The proliferative zones lining the ventricular surface generate the neurons that migrate to their proper position guided by scaffolds formed by the transient extended fibers of the radial glial cells [4]. The radial glia cells define a radial unit consisting of a relative constant number of neurons in all mammalian species investigated [1,5-7]. However, the number of radial units is increased throughout mammalian evolution. This increase in neuronal number is accomplished through extension of the time of neurogensis and decreasing the rate of neuronal death [3,8]. Furthermore, in higher mammals the proliferative zones are expanded into structured subventricular zones (SVZs) enabling them to further amplify the neuronal number [6,9,10]. The neuronal output is increased through generation of intermediate progenitor cells (IPC) that divide symmetrically in the SVZ and generate either two neurons or two IPC and thereby amplify the number of neurons [11,12].

The expansion of the mammalian brain during evolution is orchestrated with development of sulci and gyri, the convoluted folds of the cerebral cortex [13]. The process of cortical convolution is still poorly understood. The remarkable similarity of the gyration patterns among members within a species but with different patterns between species indicates that the convolution is a highly programmed process. Recent work suggested that the sites of gyral and sulcal formation can be predicted from the size of the SVZ [14]. The increase in primate SVZ complexity can, however, not solely explain the evolution of a gyrencephalic cortical surface since both lissencephalic and gyrencephalic brain structures are represented in diverse mammalian groups, including primates and rodents [6,15]. Another hypothesis suggests that mechanical forces exerted by axonal fibers are generating the gyrencephalic cortical structure [16,17]. The axonal and radial fibers attaching the growing cerebral cortex to the centre of the developing brain represent elastic elements in which the plasticity changes during elongation. Hence, the strongly interconnected cortical regions are pulled together resulting in gyri formation whereas weakly connected regions drift apart generating sulci [17,18].

Mutations causing lissencephaly, smooth brain, are identified in genes involved in neuronal migration suggesting that this step in brain development indeed constitutes a major determinant in establishment of the convoluted structure of the cerebral cortex. The first identified gene involved in human lissencephaly was encoding for the β-subunit of platelet-activating factor acetylhydrolase 1b, also known as lissencephaly 1 (LIS1) [19]. Doublecortin (DCX) was identified as the gene responsible for X-linked lissencephaly in males and subcortical heterotopia in females [20]. Mutations in the human Reelin gene are associated with recessive lissencephaly with cerebellar hypoplasia [21]. Altogether a large set of genetic data imply that cytoskeletal rearrangements and neuronal migration are crucial to development of gyrencephalic brains.

### The pig brain as a model for mammalian brain gyration

To study the process of brain gyration the pig brain constitutes an attractive alternative model to more classical laboratory animals. The convolution of the pig brain is occurring in the period between embryonic day E60 and E80 of the 117 day gestation period and the relative developmental timing and the anatomical structure is comparable to the primate brain (Nielsen et al. unpublished results and Additional file 1). At the microscopic level the process of neuronal migration shows a pronounced similarity between primates and the pig and the neuronal proliferating layers and number of migrating neurons are more prominent at E60 compared to E80 (Nielsen et al. unpublished results).

**Figure 1:**
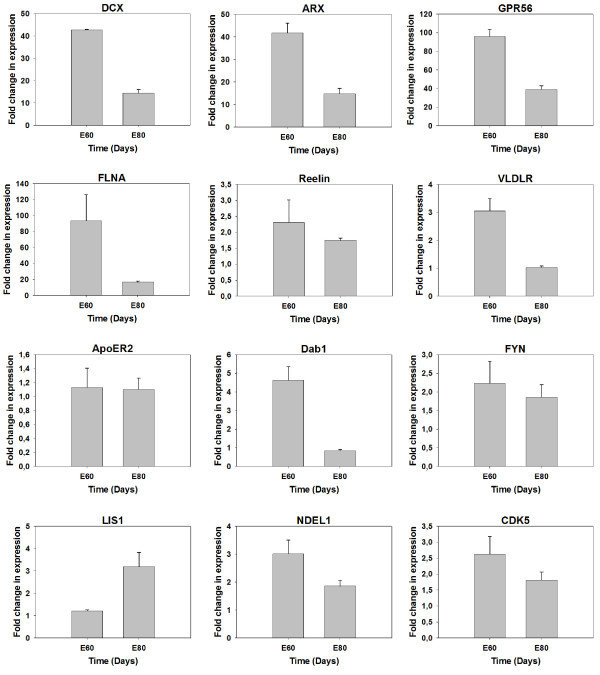
**Expression analysis of genes involved in mammalian brain convolution**. mRNA was extracted from pig cortical tissue from E60 and E80. qRT-PCR analysis were performed on cDNA for the genes DCX, ARX, GPR56, FLNA, Reelin, VLDLR, ApoER2, Dab1, FYN, LIS1, NDEL1, and CDK5. The expression levels were normalized to GAPDH, Beta-actin and 18S rRNA expression using the geNorm program [30].

We questioned which genes are differentially expressed between E60 and E80 and accordingly are candidate genes to be involved in brain development during this time frame. First we examined if a group of genes described to be involved in human brain convolution were differently expressed between E60 and E80 in pig cortical tissue. mRNA was isolated and by quantitative real-time PCR (qRT-PCR) the expression levels of such genes were measured. In the analysis we examined the expression of DCX, aristaless-related homeobox (ARX), G protein coupled receptor 56 isoform A (GPR56), filamin A gene (FLNA), Reelin, VLDLR, ApoER2, Dab1, FYN, LIS1, nuclear distribution element-like (NDEL1), and cyclin dependent kinase 5 (CDK5). For DCX, ARX, GPR56, FLNA, VLDLR, Dab1, and NDEL1 we observed a down-regulation of expression (Figure 1). LIS1 was the only of the genes up-regulated (Figure 1). Reelin, ApoER2, FYN, and CDK5 were not significantly up- or down-regulated (Figure 1). Examining also later cortical developmental time points supported the overall tendency in regulation Additional file 2. From the qRT-PCR expression analysis we conclude that some of the genes involved in the brain convolution process have an altered transcriptional activity during the timeframe in where convolution appears.

### Expression microarray analysis

For a comprehensive analysis of the differences in gene expression profiles in the pig cerebral cortex between E60 and E80 we utilized the Affymetrix GeneChip^® ^Pig Genome Array. This array contains 24,123 probes including 23,256 pig transcripts, which represents 20,201 pig genes [22]. Cortical RNA isolated from three pig E60 embryos and three E80 embryos were used for the microarray screening. Array data are available at Gene Expression Omnibus [GEO:GSE18467]. The inter-chip variance was small with a scale factor (SF) between 0.403 and 0.558 Additional file 3. The number of expressed genes present on each chip was very similar, 66%, in accordance with the number of genes hypothesised to be active during brain development Additional file 3[23]. Genes consistently up-regulated or down-regulated on the microarray triplets were further analysed. An arbitrary threshold was set at 3-fold before we classified a gene for differently regulated. This high threshold was selected to assure the minimization of background noise. 98 transcripts were found to be differential expressed by this stringency (Table 1 and Table 2). Six transcripts were represented more than once. Five transcripts were present two times; Glial fibrillary acidic protein (GFAP), the homeobox gene Meis1, megalencephalic leukoencephalopathy with subcortical cysts gene 1 (MLC1), Non-SMC element 1 homolog (NSE1), and proteolipid protein 1 (PLP) and one transcript was present three times; DEAD-box protein 17 (DBX17). Hence the total number of identified differential expressed genes was 91. Of the six genes represented multiple times on the microarray consistency in up and down regulation was observed.

**Table 1:** Whole pig genome microarray results.

Target Description	Public ID	Fold	Expression	Level
Calcium binding				
S100 calcium binding protein A1 (S100A1)	[BX917468]	6,72	Brain	2-3
S100 calcium binding protein A14 (S100 A14)	[CK451794]	5,81		NP
hippocalcin	[BI341922]	3,40	Brain	1
calbindin 2	[BQ601075]	3,20	Brain	2
autotaxin	[BX919892]	3,21	Brain	3
apolipoprotein E (APOE)	[NM_214308.1]	3,12	Brain	3
secretory protein LOC348174	[CN069500]	3,00	Brain	4
Protocadherin 15 precursor	[CO951943]	-5,08	Brain	3

Cytoskeleton organization and biogenesis				
tubulin, alpha 1 (TUBA1)	[CO954570]	6,33	Brain	3
nesprin (SYNE1)	[CD572116]	5,42	Brain	4
actinin, alpha 2 (ACTN2)	[CF795401]	4,30	Brain	4
Glial fibrillary acidic protein (GFAP)	[BF712769]	4,13	Brain	1
Glial fibrillary acidic protein (GFAP)	[BX671201]	3,94	Brain	1
Neurofilament heavy polypepteide 200 kDa	[CF180627]	3,74	Brain	3
h1-calponin	[NM_213878.1]	3,35	Brain	4

Transcription regulation				
Basic transcription element binding protein 1(BTEB1)	[BG382637]	3,12	Brain	4
Homeobox protein Meis1	[CO941421]	-3,18	Brain	4
Retinoic acid receptor RXR-gamma	[CF181317]	-3,20	Brain	4
Zinc finger protein IA-1(Insm1)	[BQ603242]	-3,82	Brain	2
KIT ligand (KITLG)	[NM_214269.1]	-3,92	Brain	3
Homeobox protein Meis1	[AW346968]	-4,12	Brain	4
Zinc finger protein ZFPM2	[CN159819]	-4,36	Brain	3
Transcription factor AP-2 gamma (AP2-gamma)	[BG609515]	-4,48	Brain	4
Jumonji domain containing protein 2C	[BF712754]	-6,68	Brain	4
Transcription factor AP-2 alpha (AP2-alpha)	[AJ657914]	-7,08	Brain	3

Signal transduction				
n-chimaerin	[CD572334]	13,67	Brain	2
ADP-ribosyl cyclase 1	[BP159691]	4,84	Brain	3*
Troponin C, slow skeletal and cardiac muscles (TN-C)	[BG382598]	3,64	Brain	4
histamine receptor H1	[BF710024]	3,28	Brain	4
Lipid phosphate phosphohydrolase 1 (PAP2-alpha)	[CF796129]	3,22	Brain	4
Frizzled 1 precursor (Frizzled-1)	[AJ682600]	-3,23	?	
proenkephalin	[BI181438]	-3,36	Brain	3
TGF-beta receptor type I precursor	[AF317296.1]	-3,57	Brain	4
Retinoic acid-binding protein II, cellular (CRABP-II)	[CN160216]	-3,67	Brain	4

Central nervous system development				
neurogranin	[BX675498]	4,91	Brain	2
proteolipid protein 1 (PLP)	[BQ601157]	4,87	Brain	2
proteolipid protein 1 (PLP)	[BQ601666]	4,09	Brain	2
Myelin basic protein (MBP)	[NM_001001546.1]	4,08	Brain	3
carbohydrate (N-acetylgalactosamine 4-0) sulfotransferase 8	[BF188991]	4,07	Brain	4
Enhancer-of-split and hairy-related protein 2 (SHARP-2)	[CO993157]	3,18	Brain	4
Slit homolog 2 protein precursor (Slit-2)	[BF712064]	3,06	Brain	4
protocadherin 18 (PCDH18)	[CF368788]	-3,21	Brain	3

Other genes				
ribonuclease, RNase A family, 1	[BG894750]	11,44	?	
armadillo repeat containing, X-linked 1 (ARMCX1)	[CK464699]	10,15	Brain	4
parkin isoform 1; parkin	[CO994470]	9,12	Brain	4
major facilitator superfamily domain containing 4	[BE012554]	7,09	Brain	3
collagen, type XXI, alpha 1 (COL21A1)	[BF440682]	7,08	Brain	3
Protein C20orf103 precursor	[CF180509]	6,72	Brain	2
Sus scrofa arachidonate 12-lipoxygenase (ALOX15)	[NM_213931.1]	6,05	Brain	4
phosphorylase, glycogen	[CF365721]	5,90	Brain	4*
muscle glycogen phosphorylase	[CF179951]	5,75	Brain	4*
coagulation factor V	[NM_214120.1]	4,57	Brain	4
Atrophin-1 interacting protein 1	[CO949606]	4,51	Brain	1
prominin 1	[CN163054]	4,41	Brain	3
dendrin (DDN)	[CF179783]	4,26	Brain	2-3
CUB and sushi multiple domains protein 2	[BI183640]	4,21	Brain	4
Pleckstrin and Sec7 domain containing protein 3 (Cytohesin)	[CN157336]	4,05	Brain	4
Probable serine protease HTRA4 precursor	[CK450639]	3,78	Brain	4*
Ras associated	[BI181239]	3,73	?	
Membrane protein MLC1	[CF368389]	3,53	Brain	2
Tetratricopeptide repeat protein 7B	[BQ600843]	3,43	Brain	3
T cell receptor	[AB079532.1]	3,43	?	
Aquaporin 1(AQP-1)	[CF792401]	3,40	Brain	3
Claudin-1	[BF188991]	3,38	Brain	4
Membrane protein MLC1	[AW478160]	3,34	Brain	2-3
Potential carboxypeptidase-like protein X2 precursor	[BQ604567]	3,30	Brain	4
retinaldehyde binding protein 1	[BX917253]	3,28	Brain	4
fibronectin type III domain containing 1(FSD1)	[CF366197]	3,26	Brain	1-2
syntaxin binding protein 5 (tomosyn)	[AJ666521]	3,25	Brain	4
hypothetical protein FLJ21127	[CN070291]	3,14	Brain	4
T-cell receptor beta chain C region	[AB079530.1]	3,05	?	
Zinc transporter 3 (ZnT-3)	[BI346660]	3,03	Brain	3
NYD-SP14 protein	[BI404541]	3,01	Brain	4
Collagen alpha 3(IV) chain precursor	[BX918155]	3,00	Brain	4
KIAA1333	[BF703163]	-3,00	Brain	4
Huntingtin-interacting protein 14	[BQ603863]	-3,02	Brain	4
vitelline membrane outer layer 1 homolog	[CF368035]	-3,03	?	
Epithelial V-like antigen 1 precursor	[CF787898]	-3,12	?	
Similar to insulin-like growth factor binding protein	[BQ599569]	-3,17	Brain	4
Probable RNA-dependent helicase p72 (DEAD-box protein 17)	[CF360658]	-3,21	Brain	4
Decorin precursor (Bone proteoglycan II) (PG-S2)	[BI182181]	-3,23	?	
Epoxide hydrolase 1 (Microsomal epoxide hydrolase)	[CF364362]	-3,31	Brain	3
metallophosphoesterase 1 (MPPE1)	[CO954514]	-3,39	Brain	3-4
NSE1	[AJ659310]	-3,46	Brain	3
NSE1	[AJ657499]	-3,52	Brain	3
serine/threonine protein kinase TAO1 homolog; STE20-like kinase	[BX676366]	-3,63	Brain	4
dopamine receptor D2	[BF712306]	-3,68	Brain	4
Collagen alpha 1(I) chain precursor	[AF201723.1]	-3,84	?	
Endothelial lipase precursor (EDL)	[BX915625]	-7,23		NP
Probable RNA-dependent helicase p72 (DEAD-box protein 17)	[BX668358]	-7,37	Brain	4
Probable RNA-dependent helicase p72 (DEAD-box protein 17)	[CO991338]	-10,71	Brain	4

Result of microarray analysis of differentially expressed genes with annotation in E60 and E80 cortex.

**Table 2:** Unannotated genes.

Transcript code	Public ID	Fold
Ssc.30169.1	[GenBank:CO986932]	4,01
Ssc.9938.1	[GenBank:BI402064]	3,81
Ssc.8325.1	[GenBank:BF712758]	3,75
Ssc.4142.1	[GenBank:CN155998]	3,07
Ssc.19138.2	[GenBank:AU060035]	-3,35
Ssc.19138.1	[GenBank:CF367810]	-3,89
Ssc.7399.1	[GenBank:BF712467]	-4,65

Result of microarray analysis of differentially expressed genes without annotation in E60 and E80 cortex. Transcript codes refer to the Affymetrix oligos spotted on the microarray [22]. The corresponding GenBank accession numbers are indicated along with the fold change in expression between E60 and E80.

**Figure 2:**
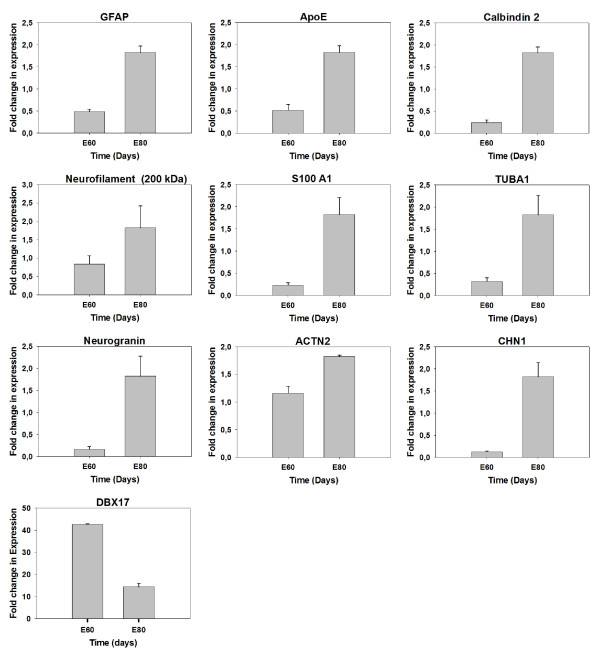
**Verification of microarray data with qRT-PCR**. Nine genes differently expressed from the microarray analysis were examined for the expression level using mRNA extracted from pig cortical tissue from E60 and E80. qRT-PCR analysis were performed on cDNA for the genes GFAP, ApoE, calbindin-2, Neurofilament (200 kDa), S100A1, TUBA1, Neurogranin, ACTN2, CHN1, and Dbx17. The expression levels were normalized to GAPDH, Beta-actin and 18S rRNA expression using the geNorm program [30].

10 of the genes differentially expressed between the two gestational time points were selected for qRT-PCR verification. These genes were GFAP, apolipoprotein E (ApoE), Calbindin 2, Neurofilament heavy chain (200 kDa), S100 calcium binding protein A1 (S100A1), Tubulin-alpha1 (TUBA1), Neurogranin, Actinin-alpha2 (ACTN2), N-chimaerin (CHN1), and DBX17 (Figure 2). For all the examined genes we observed the same tendency in regulation by the qRT-PCR analysis and the microarray analysis but the differences in fold regulation were not equal illustrating the use of two fundamental different detection approaches (see also Additional file 4). However, we conclude that an overall consistency exists between the microarray and qRT-PCR data.

The degree of annotation made available by Affymetrix covers only about 10% of the genes represented on the chip. Further annotations were accomplished through comparing the Affymetrix pig target sequences by BLAST against the Ensembl human cDNA sequence database or retrieved from the annotation list by Tsai et al., 2006 [24]. Of the identified 91 differently expressed transcripts 84 could be annotated by this method (Table 1). We note that among the annotated genes were for example TUBA1 and MLC1, both directly correlated with brain abnormalities in humans suffering of lissencephaly and macrocephaly, respectively [25-29]. Seven transcripts could not be annotated [GenBank: CO986932; BI402064; BF712758; CN155998; AU060035; CF367810; BF712467] (Table 2). Gene ontology (GO) http://www.geneontology.org annotations were determined for each individual transcript. GO terms covers the consistent descriptions of gene represented in different databases including biological processes, cellular components in which they exist, and the molecular functions they perform. The majority of the identified differentially regulated genes are highly expressed in the brain. Using biological process annotations, the genes differential expressed between E60 and E80 could be classified into groups according to calcium binding, cytoskeleton organization and biogenesis, transcription activation, signal transduction, and CNS development. A subset of the genes could not be placed in any of these functional groups.

## Conclusions

The aim of this study was to identify genes which are differentially expressed during the time of gyration of the pig cerebral cortex. It is important to notice that by screening these two time points we are not screening for genes specifically involved in brain gyration but for genes those expression is changed during the developmental and differentiation processes occurring in the time frame before and after appearance of brain gyration. We have identified for us a surprisingly low number of genes to be differently expressed between the examined embryonic time points supporting that the majority of the cortical cells have not undergone specific differentiation processes at E80 compared to E60. In this line it is important to state that neuronal migration is evident both at E60 and E80, but decreased at the latter time point. We have identified several differentially expressed genes that are described to be functional involved in neuronal migration, apoptosis, angiogenesis, myelination, and brain gyration but also a number of genes not characterised for such functions and which accordingly could be interesting new candidate genes. Further analysis will be required to determine the function of these genes during brain development.

## List of Abbreviations

ACTN2: Actinin-alpha2; ApoE: apolipoprotein E; ARX: aristaless-related homeobox; CDK5: cyclin dependent kinase 5; CHN1: N-chimaerin; DBX17: DEAD-box protein 17; DCX: Doublecortin; E60: embryonic day 60; E80: embryonic day 80; FLNA: filamin A gene; GFAP: Glial fibrillary acidic protein; GO: Gene ontology; GPR56: G protein coupled receptor 56 isoform A; IPCs: intermediate progenitor cells; LIS1: lissencephaly 1; MLC1: megalencephalic leukoencephalopathy with subcortical cysts gene 1; NDEL1: nuclear distribution element-like; NSE1: Non-SMC element 1 homolog; PLP: proteolipid protein 1; qRT-PCR: quantitative real-time PCR; S100A1: S100 calcium binding protein A1; SF: scale factor; SVZs: subventricular zones; TUBA1: Tubulin-alpha1;

## Competing interests

The authors declare that they have no competing interests.

## Authors' contributions

KBN carried out the molecular genetic and bioinformatics studies and drafted the manuscript. MK carried out the array experiments and participated in the array data analysis. IEH, ALJ, and ALN participated in the design of the study, in the evaluation of the results, and in drafting and finalizing the manuscript. All authors read and approved the final manuscript.

## Supplementary Material

Additional file 1 **Lissencephalic and gyrencephalic cortical structure during pig embryogenesis**. Gross appearance of the pig brain during development at E60 and E80. Click here for file

Additional file 2 **qRT-PCR analysis of selected genes representing all three trimesters of pig cortical brain development**. qRT-PCR analysis of a group of selected genes at cortical developmental times E40, E60, E80, E100, and E115. The examined time points are indicated. The expression levels were normalized to GAPDH, Beta-actin and 18S rRNA expression using the geNorm program. Click here for file

Additional file 3 **Inter-Chip Comparison**. The Scaling factor (SF) measures the differences in signal intensity between the individual chips. Affymetrix recommends that the SF value should be within 3-fold of one another. The number of genes expressed in cortex tissue is ~66% of the total gene number represented on the chip. Click here for file

Additional file 4 **Verification of chip data by qRT-PCR**. Comparison of fold change in expression between E60 and E80 obtained from microarray and qRT-PCR analysis. Click here for file
