# Supplementary figures and images for: 1Identification of genes differentially expressed in the embryonic pig cerebral cortex before and after appearance of gyration
Source: BMC Res Notes. 2010 May 5;3:127. doi: 10.1186/1756-0500-3-127 (PMC2877059; doi:10.1186/1756-0500-3-127)

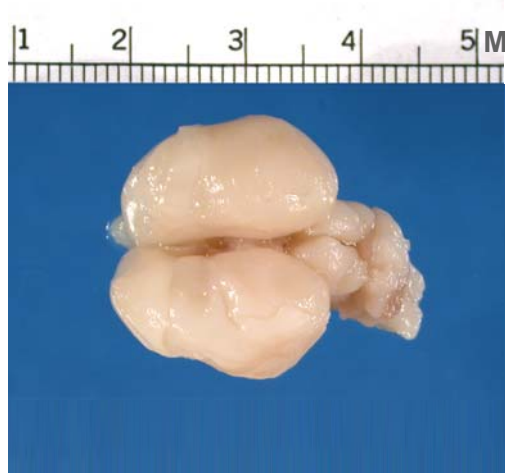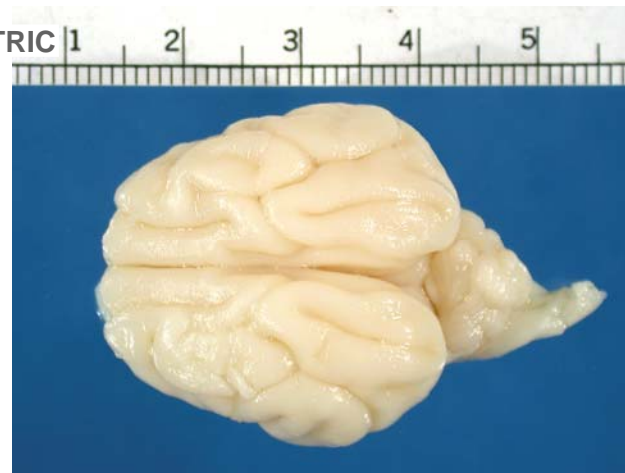

Supplement: Additional file 1 — Lissencephalic and gyrencephalic cortical structure during pig embryogenesis. Gross appearance of the pig brain during development at E60 and E80. [file 1756-0500-3-127-S1.PDF]

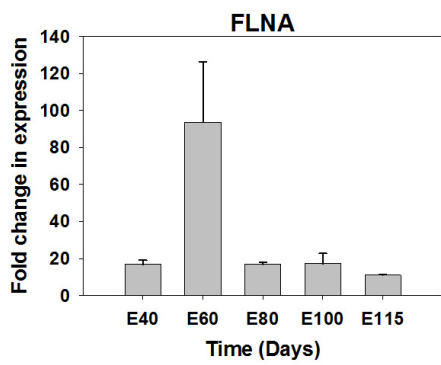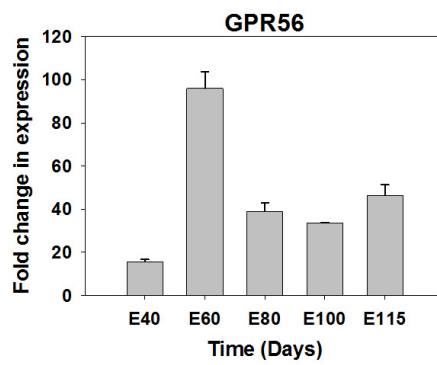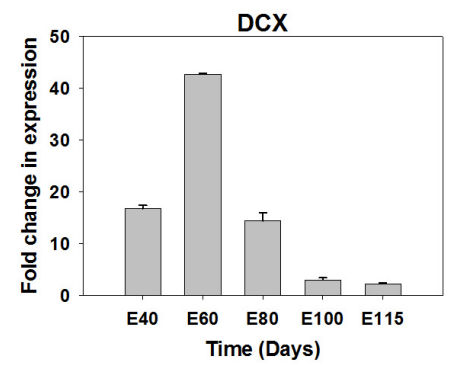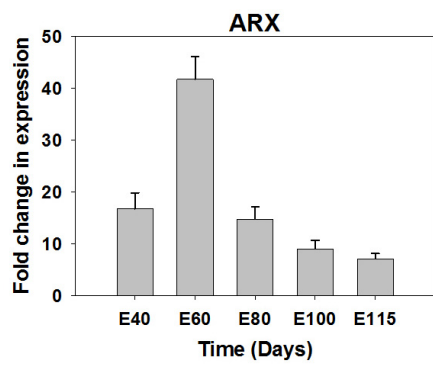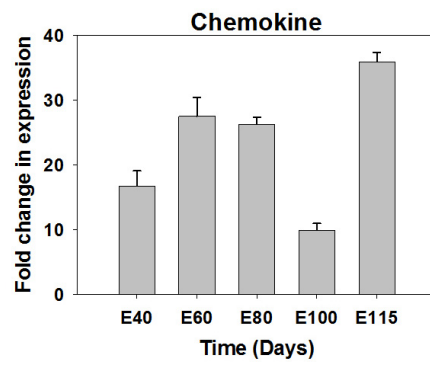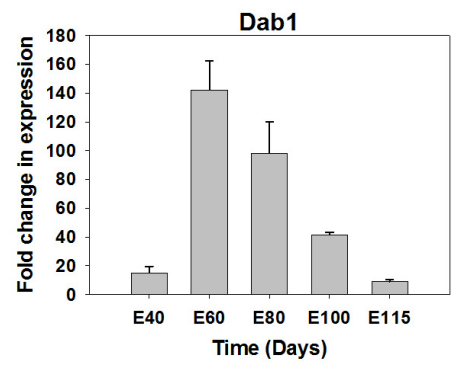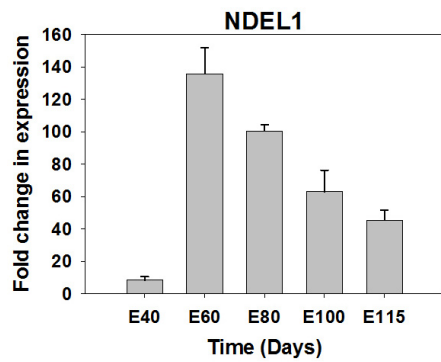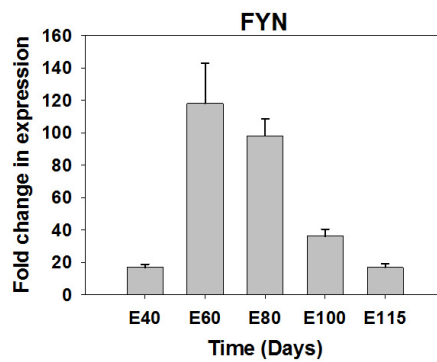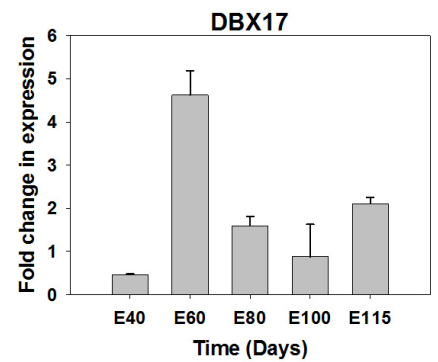

Supplement: Additional file 2 — qRT-PCR analysis of selected genes representing all three trimesters of pig cortical brain development. qRT-PCR analysis of a group of selected genes at cortical developmental times E40, E60, E80, E100, and E115. The examined time points are indicated. The expression levels were normalized to GAPDH, Beta-actin and 18S rRNA expression using the geNorm program. [file 1756-0500-3-127-S2.PDF]
