# Supplementary material for: 1Identification of genes differentially expressed in the embryonic pig cerebral cortex before and after appearance of gyration
Source: BMC Res Notes. 2010 May 5;3:127. doi: 10.1186/1756-0500-3-127 (PMC2877059; doi:10.1186/1756-0500-3-127)
Supplement: Additional file 3 — Inter-Chip Comparison. The Scaling factor (SF) measures the differences in signal intensity between the individual chips. Affymetrix recommends that the SF value should be within 3-fold of one another. The number of genes expressed in cortex tissue is ~66% of the total gene number represented on the chip. [file 1756-0500-3-127-S3.PDF]

	M114-01.CHP	M114-02.CHP	M114-03.CHP	M114-04.CHP	M114-05.CHP	M114-06.CHP
**Array Type**	Porcine	Porcine	Porcine	Porcine	Porcine	Porcine
**Embryo. Day**	**60**	**60**	**60**	**80**	**80**	**80**
**SF**	**0.558**	**0.403**	**0.555**	**0.484**	**0.55**	**0.507**
**#Probe Sets**	24123	24123	24123	24123	24123	24123
**#Present**	16379	16444	15981	16026	15916	16126
**%Present**	**67.9**	**68.17**	**66.25**	**66.43**	**65.98**	**66.85**
