# Supplementary material for: 1Identification of genes differentially expressed in the embryonic pig cerebral cortex before and after appearance of gyration
Source: BMC Res Notes. 2010 May 5;3:127. doi: 10.1186/1756-0500-3-127 (PMC2877059; doi:10.1186/1756-0500-3-127)
Supplement: Additional file 4 — Verification of chip data by qRT-PCR. Comparison of fold change in expression between E60 and E80 obtained from microarray and qRT-PCR analysis. [file 1756-0500-3-127-S4.PDF]

Gene	Fold change	
	Microarray	qRT-PCR
GFAP	4,12*	3,78
APOE	3,12	3,52
CALB2	3,20	7,73
NF-H	3,74	2,17
S100 A1	6,72	7,91
TUBA1	6,33	5,72
NGRN	4,91	11,18
ACTN2	4,30	1,57
CHN1	13,67	14,09
